# Rapid confocal imaging of vesicle-to-sponge phase droplet transition in dilute dispersions of the C_10_E_3_ surfactant

**DOI:** 10.1038/s41598-019-38620-9

**Published:** 2019-02-19

**Authors:** André Pierre Schroder, Jérôme Joseph Crassous, Carlos Manuel Marques, Ulf Olsson

**Affiliations:** 1 Institut Charles Sadron, Université de Strasbourg, CNRS UP22, F-67200 Strasbourg, France; 2 Institute of Physical Chemistry, RWTH Aachen University, D-52074 Aachen, Germany; 3 Physical Chemistry, Department of Chemistry, Lund University, SE-22100 Lund, Sweden

## Abstract

The lamellar-to-sponge phase transition of fluorescently labelled large unilamellar vesicles (LUVs) of the non-ionic surfactant triethylene glycol mono n-decyl ether (C_10_E_3_) was investigated *in situ* by confocal laser scanning microscopy (CLSM). Stable dispersions of micrometer-sized C_10_E_3_ LUVs were prepared at 20 °C and quickly heated at different temperatures close to the lamellar-to-sponge phase transition temperature. Phase transition of the strongly fluctuating individual vesicles into micrometre-sized sponge phase droplets was observed to occur via manyfold multilamellar morphologies with increasing membrane confinement through inter- and intra- lamellar fusion. The very low bending rigidity and lateral tension of the C_10_E_3_ bilayer were supported by quantitative image analysis of a stable fluctuating membrane using both flicker noise spectroscopy and spatial autocorrelation function.

## Introduction

The self-assembly of surfactants and lipids has been studied intensively for over a century^1–3^, partly because of its important role in numerous applications^4^, but also because of its direct relevance for bio-membranes^5^ and for many biological processes^6^. Aqueous surfactant systems typically display a rich phase behavior with different liquid and liquid crystalline phases^7,8^. Phase equilibria depend on local intermolecular interactions in the surfactant monolayer, resulting in its spontaneous or preferred curvature, *H*_0_, and on colloidal interactions between aggregates. Surfactant films were extensively modeled as flexible curved elastic surfaces for the investigation of microemulsions, bicontinuous cubic and sponge phases, lamellar phases and vesicles^9–12^.

**Figure 1:**
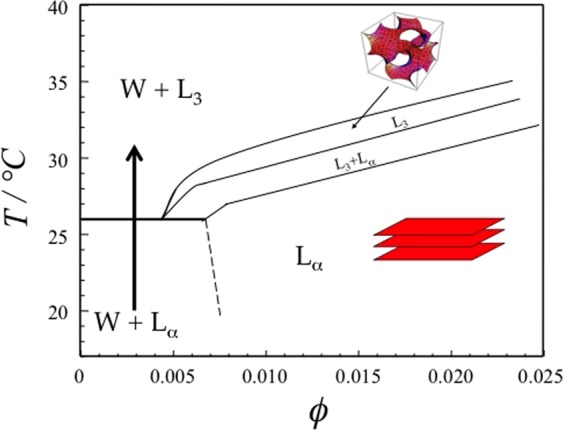
Partial phase diagram, at high water content, of the water-*C*_10_*E*_3_ system adapted from^15^. Here, *L*_*α*_ refers to lamellar phase, *L*_3_ to a “sponge” (*L*_3_) phase and W to a solution phase. *ϕ* denotes the surfactant concentration. The horizontal line at 26 °C corresponds to a three phase line, *W* + *L*_*α*_ + *L*_3_. The vertical arrow illustrates a typical temperature jump.

The monolayer curvature *H*_0_ of non-ionic ethylene oxide based surfactants, C_*i*_E_*j*_ (CH_3_(CH_2_)_*i*−1_(OCH_2_CH_2_)_*j*_OH), can be conveniently tuned by temperature making them very appealing model systems for the study of phase transitions^13,14^. Counting curvature away from water as positive, *H*_0_ decreases with increasing temperature, approximately by 10^−3^ Å^−1^ K^−1^ for triethylene glycol mono n-decyl ether C_10_E_3_^13,14^. The low surfactant concentration part of the phase diagram of C_10_E_3_-water is shown in Fig. 1. Around *H*_0_ ≈ 0, bilayer structures are preferred and the binary phase diagram shows a lamellar phase (*L*_*α*_) and a sponge phase (*L*_3_), both of which coexisting with a dilute solution phase (*W*) at high dilution. In the *W* + *L*_*α*_ two phase region, the lamellar phase can often be fragmented into unilamellar vesicles^15^. The bending rigidity of the C_10_E_3_ bilayer was determined to be 5 *k*_*B*_*T* at room temperature^16^. Vesicles, lamellar phase and sponge phase represent different topologies of the bilayer. Transitions between these states require bilayer fusion or fission, that can be triggered for such non-ionic system by temperature jump experiments. Kinetics of both *L*_*α*_-to-*L*_3_ and *L*_3_-to-*L*_*α*_ phase transitions have been studied by time resolved NMR and small angle neutron scattering^17,18^. Fusion kinetics has also been studied by temperature jumps within the *L*_3_ phase^19^. These studies concluded that membrane fusion requires essentially *H*_0_ < 0 and that the rate of fusion increases with decreasing *H*_0_. This picture was later confirmed by a vesicle stability study (Fig. 1)^15^. Extruded unilamellar vesicles, of average radius 25 nm, were found to be kinetically stable for temperatures below the three phase line *W* + *L*_3_ + *L*_*α*_, while above this temperature the spontaneous fusion occurs with a rate that increases with increasing temperature. The three phase line temperature corresponds approximately to the temperature where *H*_0_ changes sign^20,21^.

This dependence of the fusion kinetics on *H*_0_ is in agreement with previous experimental work on biological lipids and models of membrane fusion, involving a hemifusion stalk intermediate of negative monolayer curvature^22–25^. Studies on lipid systems have shown that fusion kinetics depends on the membrane composition. Lipids having *H*_0_ > 0, like single-chain oleoyl-lysophosphatidylcholine, prevent fusion, while lipids having *H*_0_ < 0, like dioleoyl phosphatidylethanolamine promote membrane fusion.

If large enough vesicles can be prepared, they can be studied and characterized with optical microscopy^26,27^. In this paper we further characterize the fusion of vesicles and the topological transitions of C_10_E_3_ membranes with temperature. Using rapid laser scanning confocal microscopy, we follow how fluorescently labeled large unilamellar vesicles (LUVs) spontaneously fuse upon a temperature jump (hereafter named T-quenching, or quenching), following the vertical line in Fig. 1, *i*.*e*. crossing the horizontal separation line (horizontal three phase line *W* + *L*_*α*_ + *L*_3_) between the *W* + *L*_*α*_ and the *W* + *L*_3_ regions. Final equilibrium structures vary from large multilamellar to droplet-like sponge phase objects depending on the quenching temperature. The sponge phase droplets formed here are analogous to the high genus vesicles studied by Noguchi^28,29^ and may also serve as a model for certain cell organelles^30,31^. Furthermore, flicker noise spectroscopy and spatial autocorrelation were applied to analyze the shape fluctuations of an isolated C_10_E_3_ bilayer, allowing us to estimate the bilayer bending rigidity *κ* and the membrane internal tension *σ*_0_.

## Results

### Imaging structural transformation

The vesicles are formed in the *W* + *L*_*α*_ two phase region where the vesicles are expected to coexist with C_10_E_3_ monomers (Fig. 1). The monomer solubility, *i*.*e*. critical aggregation concentration of C_10_E_3_ is ${\varphi }_{CAC}\sim {2.10}^{-4}$ at 25 °C^32^, corresponding to ~5% of the overall surfactant.

**Figure 2:**
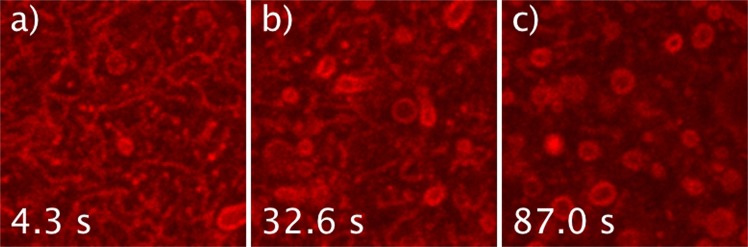
Typical evolution of a C_10_E_3_ solution that has experienced shearing during sample preparation. Images are taken from Supplementary Movie M2, during quenching at 30 °C. The solution is initially composed of tube-like structures, that evolve towards LUVs of average size of about 2 *μ*m. The characteristic tube-to-LUV relaxation time decreases with T-quenching temperature. Similar shape relaxations also take place at room temperature, over ~30 min. Image width is 19.3 *μ*m.

At 20 °C the C_10_E_3_ dispersion contains mainly stable, large unilamelar vesicles (LUVs) of ~2–3 *μ*m diameter (Fig. 2c and Supplementary Movie M1). That these LUVs exhibit high amplitude thermal fluctuations is consistent with (**i**) a reduced volume, $v=\frac{V}{4\pi {R}^{3}/3}$ smaller than one, *V* and *R* being the actual inner volume of the vesicle and the average radius of its apparent, equivalent sphere respectively, with (**ii**) a low bending rigidity of the C_10_E_3_ bilayer, *i*.*e*. $\kappa \simeq 5\,{k}_{B}T$^16^, and with (**iii**) a low enough value of the membrane lateral tension.

**Figure 3:**
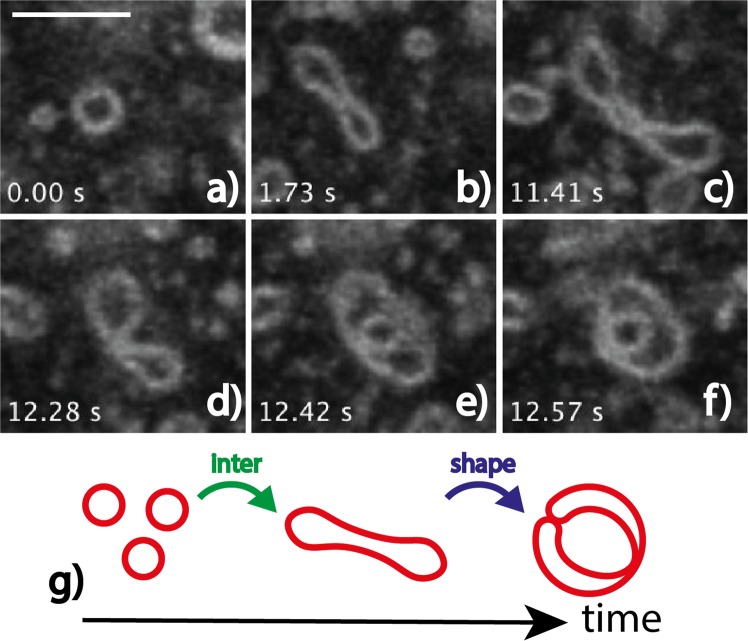
(**a**–**f**) Initial steps of multilamellar structure formation, here captured during T-quenching at 35 °C (image sequence extracted from Supplementary Movie M3). Similar evolutions of initial LUVs were observed during any T-quenching experiment (28, 30, and 35 °C, see Supplementary Movies M4–M8). (**g**) Scheme of the shape evolution with time: ‘inter’ is for inter-object fusion, and ‘shape’ for change-of-curvature-induced shape change. Time t = 0 s is arbitrary chosen. Scale bar is 5 *μ*m.

**Figure 4:**
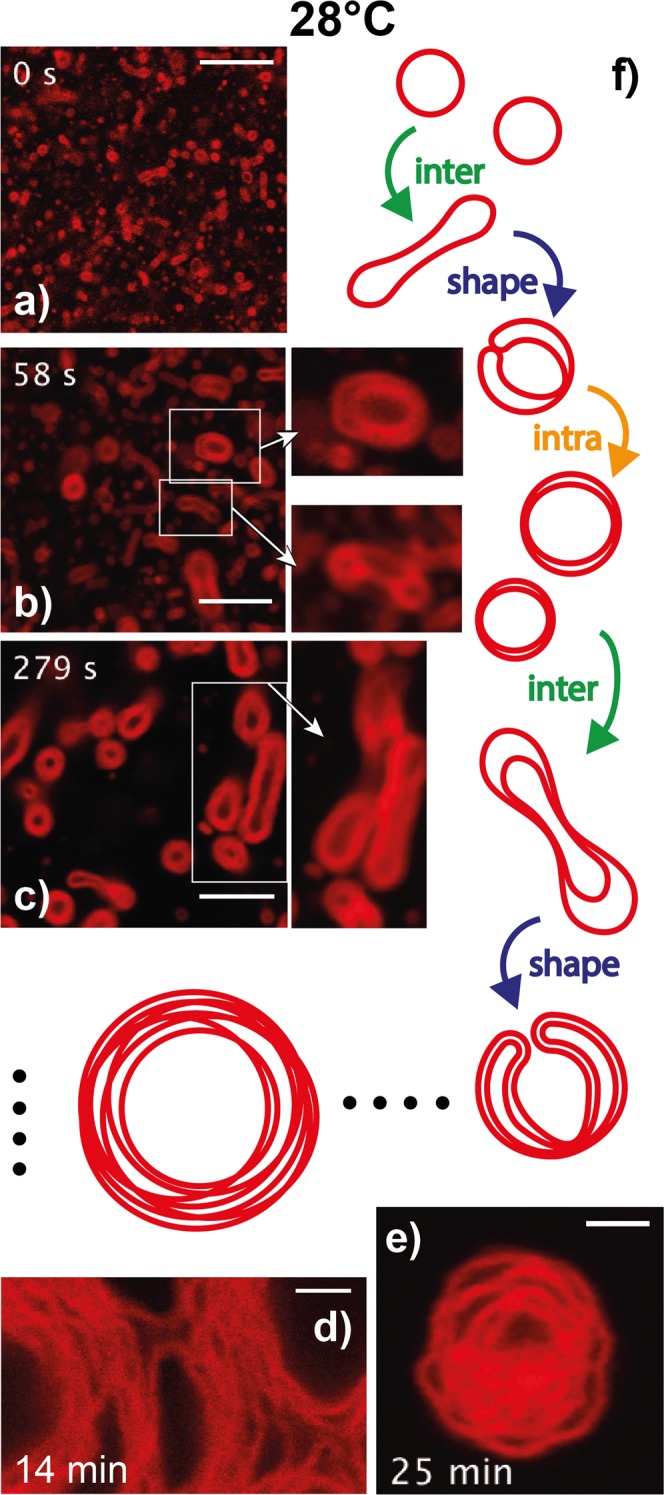
Membrane structures captured during T-quenching at 28 °C. (**a**–**c**) Evolution of initial LUVs into (apparently) bilamellar or trilamellar structures. (**d**–**e**) Equilibrated multilamellar, onion-like structures, exhibiting no or very slow structure evolution. (**f**) The self-assembly process and structural evolution are schematically depicted in steps, including ‘inter’-particle fusion, ‘shape’ transformation, and ‘intra’-particle fusion. Scale bars are 10 *μ*m in (**a**–**c**) and 2 *μ*m in (**d**,**e**).

**Figure 5:**
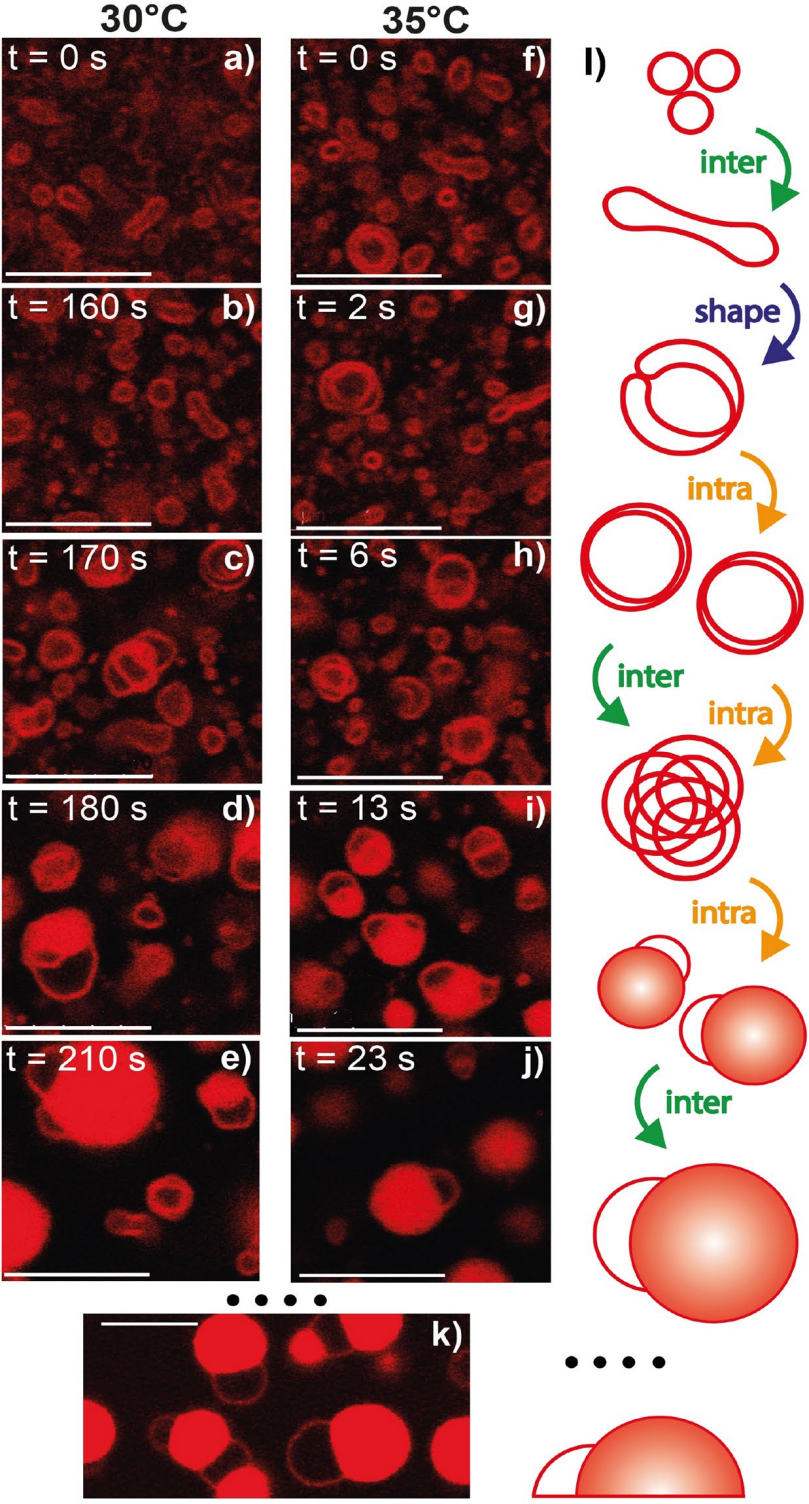
Structures captured during T-quenching at 30 and 35 °C. (**a**,**f**) Initial vesicles. (**b**–**e**,**g**–**j**) Transient bilayer structures described in Fig. 3 and their further evolution towards final, droplet-like, *L*_3_ structures. (**k**) *L*_3_ droplets adsorbed on the bottom glass surface, observed several minutes after their formation. (**l**) The schematics depicts probable steps of structure evolution, with a strong competition of ‘intra’- and ‘inter’-particle fusion, leading to ~*μ*m-sized *L*_3_ droplets (see Supplementary Movies M6 and M8). Scale bars are 10 *μ*m.

**Figure 6:**
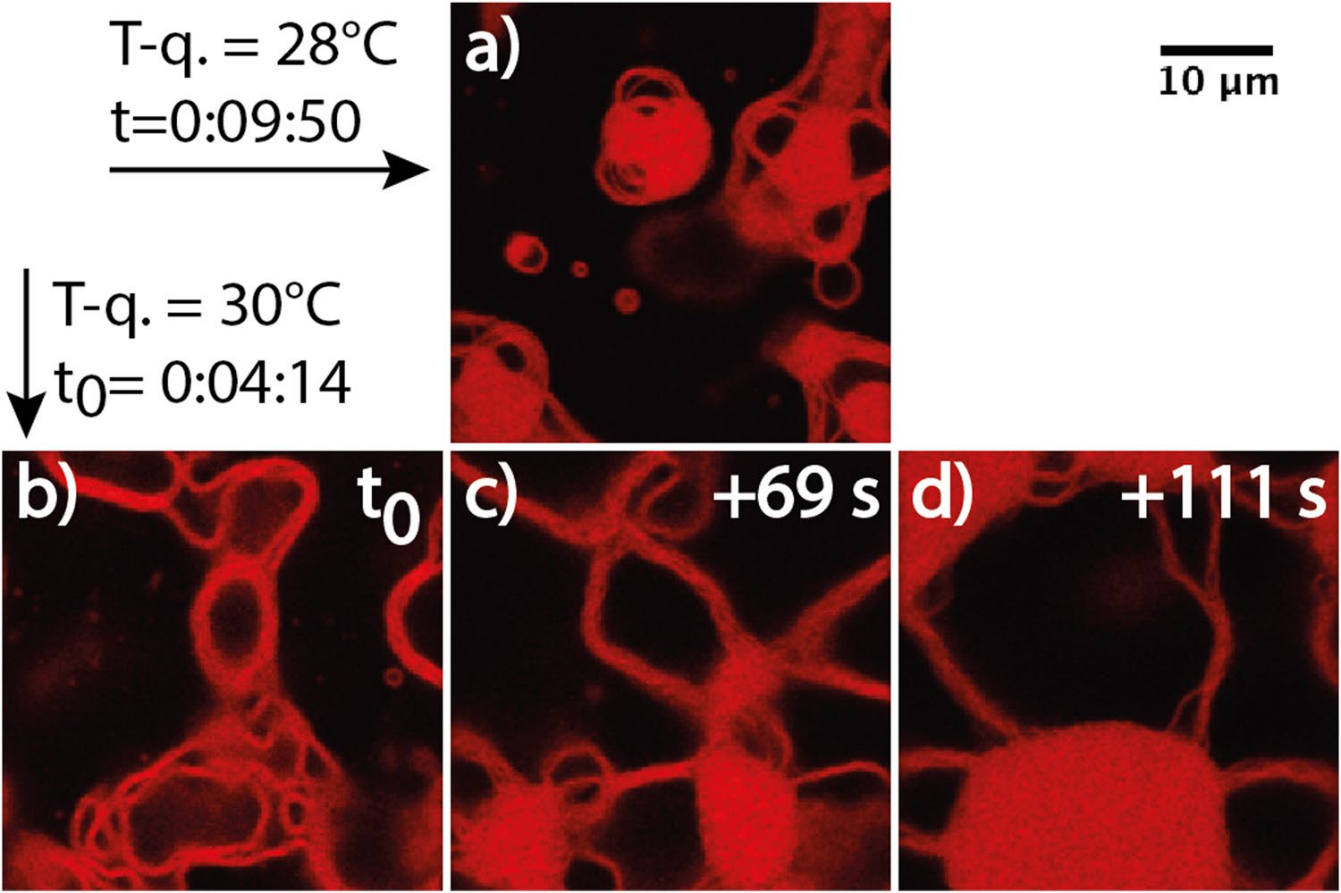
Rare objects found at 28 °C (**a**) and 30 °C (**b**–**d**). While objects in (**a**) are stable over minutes (Supplementary Movie M14), objects in the sequence (**b**–**d**) have been captured during a continuous fusion process, ending into optically dense droplets of multiply connected bilayers, as in the macroscopic *L*_3_ phase (Supplementary Movie M12).

When quenched at 28, 30, or 35 °C, LUVs undergo shape and structure transformations that we describe in the following. T-quenching experiments were repeated 3 to 5 times, Figs 2, 3, 4, 5 and 6 retain some of the typical structures observed during these experiments. When tubular vesicles induced by shearing during sample preparation (Methods section) are present in the initial dispersion, they systematically evolve towards LUVs (Fig. 2) prior to any other kind of membrane shape evolution. Such process takes several tens of seconds to a few minutes, depending on the quenching temperature, but it also takes place at 20 °C, over a longer time, *i*.*e*. ~30 min.

Figure 3 shows a typical transformation under T-quenching, of an initial, at equilibrium LUV, a process that takes ten to twenty seconds. A unilamellar vesicle first grows in size while elongating (Fig. 3b,c), and then curves until closure (Fig. 3d–f), forming a ‘bilamellar’ structure. Images in Fig. 3, extracted from Supplementary Movie M3, correspond to optical z-cuts taken at a fixed z-position in the sample. Movies of such vesicle evolution were also acquired while cycling over the z-position, refered to as (z, t) scans, see Supplementary Movie M4 that is a 3D averaging projection of such scan. They strongly suggest that the intermediate, elongated structures similar to those in Fig. 3b–d have a biconcave disk shape, while the final object in Fig. 3f, exhibits average spherical symmetry (Supplementary Movie M4). Insets in Fig. 4b (T-quenching 28 °C) further confirm this assumption. Thus, the most likely scenario for the initial LUV transformation upon T-quenching consists of a growth towards a “red blood-cell”-like structure followed by an invagination, leading to an onion-like object (Fig. 3g). We attribute the process of membrane area growing in Fig. 3b,c to membrane fusion, referred to as ‘inter’ (-object fusion) in Figs 3, 4 and 5. Indeed, our observations exclude a significant contribution of a membrane-enrichment process via the solubilized fraction of the C_10_E_3_ molecules, that would require a simultaneous decrease in size of neighboring LUVs in the sample similarly to the Ostwald ripening process^33^, a phenomenon never observed in our experiments (see Supplementary Movies M4–M8). Fusion events of small LUVs could not be resolved, we could however catch some fusion events of multilamellar objects. Besides, the shape evolutions shown in Figs 3c–e, 4 and 5, corresponding to the ‘shape’ transition schematically depicted in the figures, is compatible with the expected tendency of the C_10_E_3_ bilayers to form saddle shape structures (invagination process) while temperature increases in the sample, to accommodate for the concomitant decrease in *H*_0_. Objects similar to Fig. 3f were seen to only persist over short times (several seconds), before undergoing further structural transformations, leading to either larger and topologically more complex multilamellar objects, or denser *L*_3_ phase droplets.

When quenched at 28 °C, the initial unilamellar vesicles mainly transform into larger multilamellar *L*_*α*_ structures through repeated sequences of growth, anisotropic deformation, fusion, invagination, leading to objects often presenting more than ten apparent membrane layers. Stable structures were obtained after 3 to 8 min, keeping in mind that stability is considered over an experimental time scale of ~30 min. Typical stable multilamellar structures are shown in Fig. 4d,e, being whether onion-like, *i*.*e*. symmetric (Fig. 4e), or non symmetric (Fig. 4d), as shown in Supplementary Movies M9–M11. On the contrary, T-quenching at 30 °C (resp. 35 °C) results after a period of 2 to 5 (resp. 0.5 to 3) min in dispersions containing mainly *L*_3_ droplets capable of fusion with neighboring analogs upon contact (Fig. 5e,j, Supplementary Movies M6, M12 and M13), which eventually adsorb at the bottom coverslip of the observation chamber in less than 2 min (Fig. 5k).

Morphological transitions of the bilayers during the quenching experiments are sketched in Fig. 4f (28 °C) and in Fig. 5l (30 and 35 °C). Beside ‘inter’-object fusion and ‘shape’ transition (Fig. 3) was an ‘intra’-object fusion process clearly evidenced upon T-quenching experiments at 30 and 35 °C, while to a less extent upon T-quenching at 28 °C (Figs 4f and 5l respectively). Schematically we can describe the overall process after a temperature jump as follows. It begins with the fusion of the initial unilamellar vesicles. Such events, when they occur, are rapid and not resolved in our experiments for initial LUVs. However, we clearly see the decrease in the number density of vesicles and the increase of their average size. From these very first moments is the local temperature increase of the sample related to a decrease of *H*_0_, and hence to a bilayer topology transformation resulting in a gradual decrease of the average Gaussian curvature, 〈*K*〉, and the Euler characteristic, *χ*_*E*_ = 〈*K*〉*A*_*b*_/2*π*^34^, of the membrane, where *A*_*b*_ is the bilayer membrane area. Bilayers accommodate that temperature increase with the creation of saddle points, that in turn results in the ‘shape’ transformation depicted above, *i*.*e*. in the evolution towards onion-like structures with an increased number of bilayers. As seen in Fig. 3 and in Supplementary Movies M3–M8, such ‘shape’ transformation process expands over several seconds. For a low enough temperature increase, and certainly for a low enough value of the rate of temperature increase has the system the time to evolve through the building of such multilamellar objects, where bilayers still exhibit micrometer range values of their radius of curvature. This corresponds mainly to 28 °C T-quenching experiments (Fig. 4b–e). Besides, is the rate of local temperature increase much higher upon T-quenching at 30 °C and 35 °C, concomitant to a stronger and faster decrease of *H*_0_. As a consequence, bilayers certainly cannot undergo the required rate of sequences of ‘inter’-fusion and ‘shape’-transformation in order to accommodate with the corresponding high number of saddle points. The system thus rearranges through ‘intra’-fusion processes, leading to a denser organisation of the bilayers, with sub-micron range values of their local radius of curvature. This gradual topology transformation and increase of the bilayer volume fraction, *ϕ* of the structures, is expected to terminate when *ϕ* reaches the value at the sponge phase binodal line to the given temperature. From the phase diagram, we thus expect the steady state *ϕ* in the sponge phase droplets to increase with increasing temperature. Our experiments show that a wide variety of structures coexist during T-quenching. As a matter of fact, it can be considered that a competition between inter-vesicle and intra-vesicle fusion is taking place, controlled by the local rate of temperature increase. Thus, that variety of structures found is likely the result of that competition. An example of such variety is given in Fig. 6. One the one side, at 28 °C, amongst major onion-like structures, were found some rare, denser structures that did not fuse at the experimental time scale (Fig. 6a, Supplementary Movie M14), while at 30 °C, amongst major *L*_3_ droplets, were found big, space extended structures, similar to the ones shown in Fig. 6a, but with a strong fusogenic character (Fig. 6b–d, Supplementary Movie M12).

We note that the sponge phase droplets often contain one or more unilamellar membrane “domes”, as seen in Fig. 5e,j. Certainly, dome precursors are seen in Fig. 6a and Supplementary Movies M14 and M15. While in those, stable intermediary structures (28 °C), multiple bilayers are observed, this is rarely the case in *L*_3_-phase droplets, *i*.*e*. at 30 and 35 °C (Fig. 5e,j), where most often only one dome remains per droplet. Similar domes are also often observed on cubosomes^35^, that could be seen as the crystalline analogs of sponge phase droplets. In the bicontinuous cubic phase the multiply connected bilayer has crystallized in a cubic lattice, while being disordered (liquid) in the case of the sponge phase. In both cases however, the multiply connected bilayer has to meet droplet interface in a particular way to avoid unfavorable bilayer edges. Confined between two solid surfaces, a sponge phase was found to form planar lamellae^36^. Hereby, the sponge phase droplets are pictured as having a unilamellar bilayer envelope, from which the multiply connected sponge structure grows inward as it necks in the envelope. Surprisingly, those domes remain present once *L*_3_-phase droplets have adhered on the bottom glass substrate (Fig. 5k).

### Bending modulus of a single bilayer

Mechanical properties of fluid membranes have been extensively studied over the last three decades starting with the pioneering work by Helfrich^9,37–39^. This field of research was born with the growing interest in phospholipid bilayers, that not only appeared as model systems of cell membranes, but also as interesting tools for investigating soft matter physics. Thanks to their very low solubility, typically in the order of pM to nM, phospholipids build, indeed an almost perfect system, *i*.*e*. free standing bilayers in a surfactant-free solution. Conversely, C_10_E_3_ has a non negligible monomer solubility, representing 5% of the molecules in our experiments. However, this work demonstrates that C_10_E_3_ membranes exhibit long term stability at a constant temperature, *i*.*e*. over tens of minutes, though continuously exchanging surfactant molecules with their aqueous environment.

**Figure 7:**
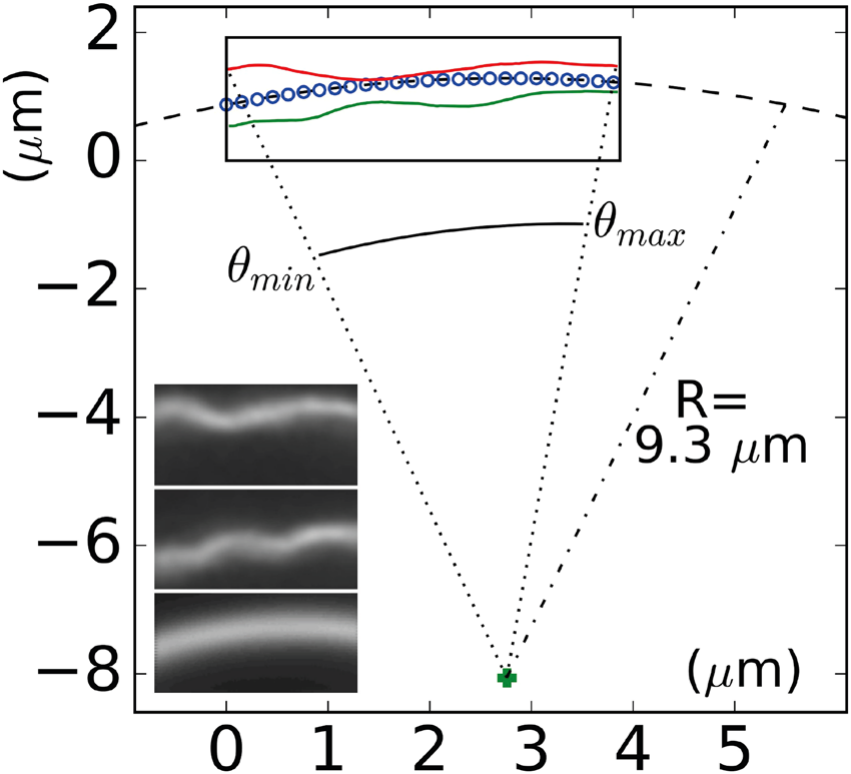
Insets from top to bottom, two snapshots of Supplementary Movie M16, and average image over the whole movie. Rectangle is the movie window (128 × 64 pixel^2^ = 3.87 × 1.92 *μ*m^2^), that contains the calculated fluorescence maximum profiles of the three fluorescence images given as insets (see SI section for the detailed procedure of profile extraction). Dotted line is the average intensity profile best fit with a circle of radius 9.3 *μ*m, green cross is the calculated center of symmetry of the circular best fit.

We imaged a fluctuating bilayer at a frame rate of 172 frames/sec, that was found to be sufficiently high to enable the resolution of the membrane fluctuations (see Supplementary Movie M16, and Fig. 7). The imaged membrane corresponded to a region of a dome from a stable multilamellar structure at 28 °C, similar to the one shown in Supplementary Movie M15. The observed section of membrane was distant enough from the rest of the structures so that no steric interactions with another membrane was observed during the movie. Images were processed as described in the SI section, in order to extract the membrane profile (*x*(*t*), *y*(*t*)) with sub-micron resolution. Figure 7 shows two typical snapshots of the Supplementary Movie M16 together with the time averaged image of the movie (grey level insets, average on bottom), as well as the corresponding extracted profiles (rectangular window). As seen in the figure, the average membrane profile is circular, with a radius of curvature *R* = 9.3 ± 0.1 *μ*m, thus showing that domes are sections of a sphere. (*x*(*t*), *y*(*t*)) profiles were further converted into circular coordinates, and expressed in terms of *r*(*θ*(*t*), *t*), the radius of the profile from the center of symmetry of the spherical dome, *θ*(*t*) being the angle variable in the cylindrical reference space (in 2D geometry in the images). Finally, we calculated the height function *h*(*θ*, *t*) between average and instantaneous membrane profiles as described in the SI section.

Following methods well described in literature, we applied flicker noise spectroscopy to the space or time variations of the *h*(*θ*, *t*) curves, to extract the bending rigidity *κ* of the membrane: Table 1 compares *κ* obtained from the analysis of the average fluctuation spectrum^40^ and from the evaluation of the average angular autocorrelation function^41^ of the membrane profile. Both values are close to the value published in literature^16^. Detailed calculations for *κ* are given in SI section.

**Table 1:** Bending modulus *κ* as calculated from spectrum analysis and autocorrelation analysis, and comparison with measured data from literature.

Bending modulus (*k*_*B*_*T* units)	literature^16^	spectrum analysis	autocorrelation analysis
*κ*	4.95 ± 0.2	1.7	3.9

## Discussion

Our study demonstrates the applicability of rapid confocal microscopy and highly fluctuating C_10_E_3_ vesicles to investigate complex membrane fusion processes *in situ* through T-quenching. Those assembly processes are driven by the *L*_*α*_-to-*L*_3_ phase transition of the surfactant, being characterized by the inter and intra fusion of suspended unilamellar vesicles (LUVs), and the formation of either large multilamellar objects or micrometric sponge phase droplets. This was made possible first, by working with dilute samples (0.003 w/w), and secondly, by quenching samples initially in the *L*_*α*_ phase at three temperatures close to and above the *L*_*α*_-to-*L*_3_ transition temperature. For the lowest temperature quenching, *i*.*e*. 28 °C, we describe how primary LUVs evolve over minutes towards multilamellar objects with increasing number of membranes and local membrane concentration. We also show more rare metastable structures, that appear as intermediates between the lamellar phase *L*_*α*_ and the *L*_3_ sponge phase, being characterized by such an increased local surfactant density that no more individual membranes can be seen with our optical technique. In comparison, temperature quenching at 30 °C or 35 °C led to much faster kinetics of membrane transformation. As a consequence, most of the intermediate structures seen at 28 °C were never observed, and LUVs were evolving over a few seconds towards the sponge phase droplets. Finally, we could record an isolated fluctuating part of a membrane at 28 °C, that we analyzed using both flicker noise spectroscopy, and spatial autocorrelation, to estimate the bending modulus *κ* of the membrane at that temperature. Our measurement of the bending modulus provides values in the range of 2–4 *k*_*B*_*T*, in good agreement with literature values^16^.

## Methods

### Vesicle solution and sample preparations

C_10_E_3_ (Sigma Aldrich) was mixed with an amphiphilic dye (BODIPY® 558/568-C12, from Life Technologies) at a dye/C_10_E_3_ fraction of 0.3 mol%. This mixture was diluted at a mass concentration 3.33 g/L in 18 MΩ deionized water (Merck Millipore, Germany), corresponding to a molar concentration 11.5 mM, and to a volume fraction *ϕ* = 0.315%, considering a density *ρ* = 0.94 g/cm^3^ ^32^. Once prepared, the dispersion was gently shaked during three minutes. Such solution showed long term stability at 20 °C, as observed under the microscope. The stock solution was used within 2 days. Observation cells were build using the following protocol: a 1 mm thick double face tape, with a 6 mm diameter pinched hole was stuck on one side of a glass coverslip, forming a cuvette, in which 28 *μ*L of the vesicle solution was injected. The well was then closed by sticking on the free tape side a 1 mm thick glass slide, resulting in a watertight observation cell. The preparation was done at room temperature, *i*.*e*. at 20 ± 1 °C. It is worth noting that C_10_E_3_ vesicles are shear sensitive. The transfer of the 28 *μ*L droplet was done using a pipette tip (Eppendorf, Germany); the sample injection eventually resulted in the shearing of the vesicles into long membrane tubes that relaxe over tens of minutes at 20 °C, and over less than five min during one typical T-quenching experiment, with a strong dependence on both jump temperature and sample preparation conditions. Such evolution is shown in Supplementary Movie M2 and Fig. 2a–c.

### Confocal microscopy

The confocal micrographs were recorded on a Leica SP5 CLSM (Germany) operated in the inverted mode (D6000I) using a 100×, 1.4 NA, oil-immersion objective. A 543 nm He-Ne laser was used to excite the amphiphilic dye. The samples were monitored *in situ* at different temperatures, and an environmental system was used to ensure temperature control with an accuracy of 0.2 °C. The microscope was equilibrated over one hour at the quenching temperature before starting the experiments. A typical T-quenching experiment ran as follows: an observation chamber filled with the LUVs was placed on the microscope stage just after it has been sealed, and the real time confocal imaging was started as soon as possible; we consider that image recording started within less than 1 min (typically 30 s) after the observation cell was placed in the microscope. For each T-quenching experiment the first image sequence was acquired at a frame rate ~10 to 30 frames/s, during 1 to 5 min, in a region of the sample located ~50 *μ*m above the coverslip. Then, other image sequences were acquired at different positions in the sample, with the purpose of collecting images on all possible structures present in the dispersion. In particular, reducing the field of view while increasing the frame rate (up to ~200 frames/s) enabled, at high magnification and resolution, to resolve the membrane thermal fluctuations.

Confocal images of lamellar and sponge phase structures are shown without modification except global image contrast enhancement and smoothing if necessary.

## Supplementary information

SI-ESI file

Movie M1

Movie M2

Movie M3

Movie M4

Movie M5

Movie M6

Movie M7

Movie M8

Movie M9

Movie M10

Movie M11

Movie M12

Movie M13

Movie M14

Movie M15

Movie M16
