# Supplementary material for: Rapid confocal imaging of vesicle-to-sponge phase droplet transition in dilute dispersions of the C10E3 surfactant
Source: Sci Rep. 2019 Feb 19;9:2292. doi: 10.1038/s41598-019-38620-9 (PMC6381169; doi:10.1038/s41598-019-38620-9)
Supplement: Supplementary file 1 — SI-ESI file [file 41598_2019_38620_MOESM1_ESM.pdf]

Rapid confocal imaging of vesicle-to-sponge phase droplet transition in dilute dispersions of the $C_{10}E_3$ surfactant.

André Pierre Schroder, Jérôme Joseph Crassous, Carlos Manuel Marques, and Ulf Olsson

1 SUPPLEMENTARY INFORMATION

1.1 Maximum fluorescence profile determination

An 'isolated' region of a connected membrane was imaged with the confocal fluorescence microscope at a rate of 172 frames per second, with the constraint that the membrane never moved out of the observation field, and always joined the two vertical borders of the image (SI.Fig. 1a, Movie ESI.M16). Window size in Movie ESI.M16 corresponds to 128×64 pixel^2, *i.e.* 3.87×1.92 μm^2. We extracted from the movie a continuous sequence of 1276 images, corresponding to ∼7.4 seconds, that we used for the analysis described below. Thanks to these (small) dimensions and (fast) acquisition frame rate, image bluring that induces a lack of precision in the membrane profile localization was minimized. We established a protocol to extract the main intensity profile of the membrane in each image, that we describe now. Similar protocols have been published in the case of round shaped profiles of GUVs, whether with phase contrast[?] or fluorescence microscopy[?] imaging.

SI.Fig. 1a is a snapshot of Movie ESI.M16. Each original image was first completed with a frame of 16 pixels width (new image size = 160×96 pixel^2), with grey values choosen as follows : upper and lower bands (16 pixels height each) were filled with a unique grey level, equal to the average grey of the first and last row of the original image. Each row of the original image was completed on its left and right side by 16 pixels with a value equal to that of the corresponding border pixel (SI.Fig 1b). The image was then smoothed by replacing each pixel value $I_{x',y'}$ by the value $I_{x,y}$ given by

$$I^{(L)}_{x,y} = \frac{\sum_{x',y'} I_{x',y'} \exp\left[-\frac{(x'-x)^2+(y'-y)^2}{L^2}\right]}{\sum_{x',y'} \exp\left[-\frac{(x'-x)^2+(y'-y)^2}{L^2}\right]}$$

with x,y (and x',y') the column and raw indices of the image. x',y' were choosen such that $\sqrt{(x'-x)^2+(y'-y)^2} < L$ (SI.Fig. 1c). The value $L = 4$ was the smallest value that blurred the image such that fluorescence intensity along each column of the image exibits a smooth pic shape, and also such that the full collection of these fluorescence maxima along the image width composes a continuous curve, as shown in SI.Fig. 1d. At this stage a first estimation of the membrane profile is therefore a collection of 128 maxima $M_{i(i=0..127)} = (i, y_i^M)$, with integer coordinates (SI.Fig 1g, empty squares). The following procedure applied to each M_i reads to sub-pixel resolution: five, 21 pixels long, intensity lines centered on M_i, along i) the vertical (Oy) direction ($\phi = 0$), and along ii) the directions oriented with angles $\phi = 22.5°, 45°, -22.5°, -45°$ with respect to the (Oy) axis, were extracted, as shown in SI.Fig. 1e,f. Each of the five intensity data sets were then fitted with a quadratic function, $I_i^\phi = a_i^\phi * dl_\phi^2$, dl_ϕ being the displacement variable along the ϕ direction (SI.Fig. 1f). The maximum of each fitting line was then converted into subpixel, *i.e.* real number coordinates $(x^M_{i,\phi}, y^M_{i,\phi})$. Not each of the five coordinates had how-

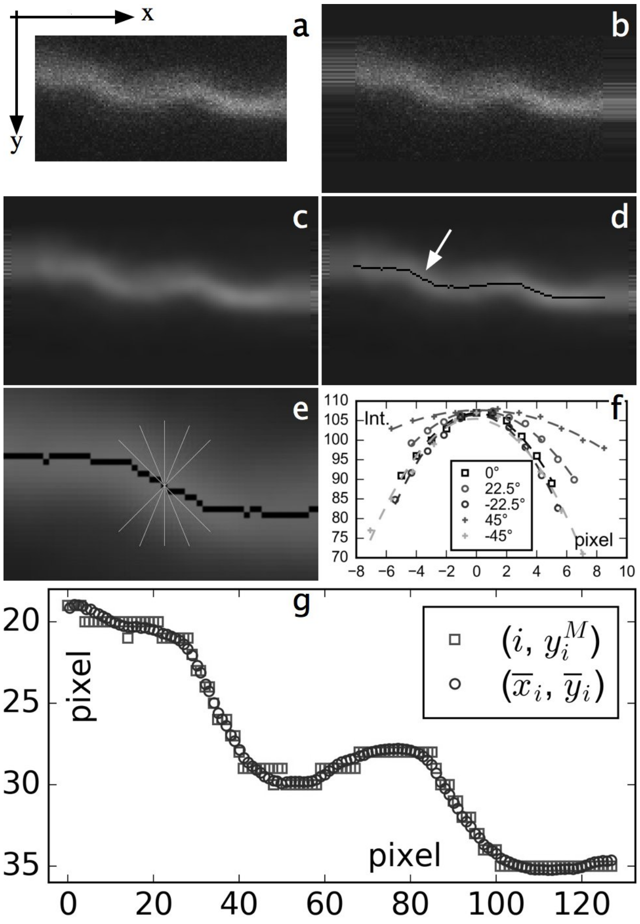

Fig. 1 : a) Snapshot of Movie ESI.M16. An isolated, but still connected to a bigger multilamellar structure (image not shown), membrane is captured at a frame rate of 172 frames/sec; b) original image is extended with a frame of width 16 pixels; c) smoothing of b) as described in the text; d) maximum intensity profile of the membrane, as obtained by highlighting the highest intensity pixels in each pixel column; e) zoom of d) arround the white arrow in d) and scheme of the 5 lines over which the fluorescence intensity is extracted and analyzed; f) the five intensity lines for the five lines in e), 0° corresponds to the vertical direction, white pixel would take the value 255; g) pixel (□) and sub-pixel (○) resolution maximum fluorescence intensity profiles for the whole membrane shown in a).

ever the same level of confidence. Indeed one expects that intensity profiles with a direction closer to the local membrane profile orientation are less trustable than intensity lines that are close to the normal to the local membrane profile. For each M_i, profiles with 'poor' orientation were determined as the ones with a second order coefficient of their quadratic fit intensity profile a_i^{ϕ} more than two times smaller than the highest quadratic coefficient of the set of five ϕ directions; they were ignored in the following of the procedure. Then, the center of mass $(\bar{x}_i,\bar{y}_i)$ of the remaining maxima was calculated for each M_i, by weighting each maximum position $(x^M_{i,\phi},y^M_{i,\phi})$ with the quadratic value a_i^{ϕ} of the corresponding intensity line. The final maximum intensity profile of the membrane is thus a new collection of 128 points with sub-pixel precision, a fortiori not equally spaced in the (Ox) direction. SI.Fig. 1g shows the intensity profile (i,y_i^M) (pixel resolution) and the final sub-pixel profile $(\bar{x}_i,\bar{y}_i)$ for image SI.Fig. 1a. In the following, $(\bar{x}_i,\bar{y}_i)$ profiles will be refered to as $(x_i(t),y_i(t))$, with t the time variable, corresponding equivalently to the image index, or even simply, by $(x(t),y(t))$, with the implicit decomposition of the profile into 128 discrete values, fixed by the geometry of Movie ESI.M16.

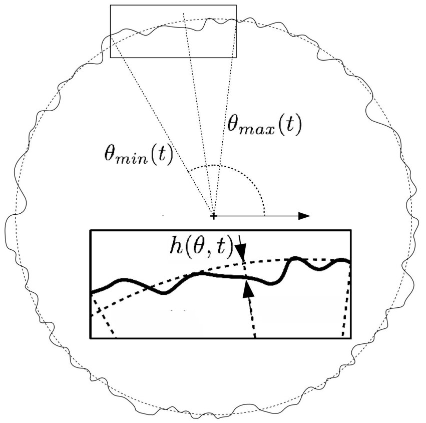

Fig. 2 : Scheme of an equatorial section of a spherical membrane, at time t. The membrane profile, originally expressed as a set of $(x(t),y(t))$ coordinates (pixels or subpixels), can be expressed into a cylindrical $(\theta,r(\theta,t))$ representation. $h(\theta,t)$ is the distance function between the membrane and it's average, round shape position (dotted curve). In the present case, due to the restricted area of observation, the instantaneous $(\theta_{min}(t),\theta_{max}(t))$ limits do not corresond to the $(\theta_{min},\theta_{max})$ limits choosen for the analysis of the whole movie (see the text).

Flicker noise spectroscopy of membranes is most frequently applied through the shape analysis of the 2D projection of 3D vesicles.[?,?,?] Averaging Movie ESI.M16 has shown that the studied membrane has here an average circular shape (Fig. 7 in main article). We thus argue that we can use a similar protocol to that reported in litterature, under suitable precautions. SI.Fig. 2 shows the equator section of a membrane with an average spherical shape. The 2D-projected membrane fluctuates overtime around it's average, circular shape due to thermal energy, with an amplitude spectrum that depends on both its bending modulus κ and its internal tension σ.[?] Following the litterature, original $(x(t),y(t))$ membrane profiles are better analyzed under the form of $r(\theta,t)$ profiles, *i.e.* the distance function between the membrane and the center of the vesicle. Implied in this cartesian to cylindrical transformation is a decomposition of the contour into a set of θ angle values regularly spaced in the $[0..2\pi]$ range, common to all images,[?,?] as far as a closed, almost circular contour is studied. However a partial, opened contour can be studied as well, as described elsewhere.[?] Let's assume one captures one region of such 2D projection, *i.e.* in a rectangular window as schematized in SI.Fig. 2. Applied to our experiment, the center of the circular best fit to the average profile (see above) has to be chosen as the center of symmetry for the cartesian to cylindrical variable conversion (Fig. 7 in main article). Each membrane profile, characterized by its 128 values $(x(t),y(t))$ defines a unique $[\theta_{min}(t)..\theta_{max}(t)]$ range (SI.Fig 2), while flicker noise spectroscopy analysis requires a unique $[\theta_{min}..\theta_{max}]$ range for the angular extension of the whole set of profiles. We define $[\theta_{min}..\theta_{max}]$ so that θ_{min} is the biggest value of the set of $\theta_{min}(t)$, and θ_{max} is the smallest value of the set of $\theta_{max}(t)$, taken over the entire movie, as illustrated in Fig. 7 in main article for the particular case of two profiles only. Please note that the so defined $[\theta_{min}..\theta_{max}]$ range is included in the $[\theta^{AV}_{min}..\theta^{AV}_{max}]$ range of the average, circular shape membrane profile (Fig. 7 in main article).

To summarize, the $(\bar{x}_i,\bar{y}_i)$ sub-pixel coordinates of each membrane profile were first converted into a $r(\theta(t),t)$ profile, and then converted against the $[\theta_0..\theta_{127}]$ unique base of angles common to all images, as already explained above; this last conversion was obtained with a usual spline interpolation. We finally introduce a new variable, h (SI.Fig 2) corresponding to the height between the instantaneous profile and the averaged one:

$$h(\theta,t) = r(\theta,t) - R \quad (1)$$

In the following we refer to the $h(\theta,t)$ distribution as $h(t)$.

1.2 Spectral analysis

The bending modulus κ and the membrane tension σ are theoretically accessible from the analysis of the spectral distribution of the membrane thermal fluctuations using the theory of Helfrich.[?,?] Consider a flat, fluctuating membrane of surface area A with small amplitude oscillations relative to its average position, *i.e.* characterized by $h(x,z) \ll \sqrt{A}$, (x,z) being the reference plane of the average membrane. Following Helfrich notation,[?] the energy cost due to both

stretching and bending can be written as:

$$F = \int dA \left[\frac{1}{2}\, \kappa\, (\nabla^2 h)^2 \, + \, \frac{1}{2}\, \sigma\, (\nabla h)^2 \right] \tag{2}$$

Spatial Fourier transformation gives the energy per q-mode, which is known to be $\frac{1}{2}k_BT$, due to the equipartition theorem. The mean square amplitude of each mode can thus be calculated as:

$$\langle h_q^2 \rangle = \frac{k_BT}{\kappa q^4 \, + \, \sigma q^2} \tag{3}$$

with $q^2 = q_x^2 + q_z^2$. Though derived for a flat open membrane, this formula can be applied to the modes of a quasi-spherical membrane, given the fact that it might be less valid for the lowest modes.?

In the particular case of vesicle observation using video microscopy, measurable membrane fluctuations are in fact that of the circular cut of the equatorial plane of the vesicle around the average membrane position, which is expected to be circular, of radius $\langle R \rangle$. Following the same notations as above, the plane of observation is then the (x,y) plane, and thanks to the cylindrical geometry of the average membrane, $h(x,y)$ profiles can further be expressed into cylindrical coordinates, becoming $h(\theta)$. As a consequence of membrane projection, the dimensionality of the problem is reduced, reading the following mean square amplitude for the membrane fluctuations? :

$$\langle h_{q_s}^2 \rangle = L \frac{k_BT}{2\sigma} \left[\frac{1}{q_s} - \frac{1}{\sqrt{\frac{\sigma}{\kappa} + q_s^2}} \right] \tag{4}$$

with $q_s = \frac{2\pi n}{L}$, and $n \in [0, N/2]$, with $L = 2\pi\langle R \rangle$, and N the number of angles over which the contour is numerized. Note that easy Fast Fourier Transform (FFT) is obtained for N taken as a power of two. As explained in the previous section, membrane profiles $h(t)$ of Movie ESI.M16 (SI.Fig. 1) have been numerized over N =128, regularly spaced values of the angle θ, and their FFT was calculated using available routines in python (https://www.python.org/). q-modes are here calculated for $\langle R \rangle = 9.3$ μm, and $L = \langle R \rangle(\theta_{max} - \theta_{min})$, as explained above. The average spectrum over the whole movie is given in SI.Fig. 3, together with its best fit using expression (4), in function of the wave vector q_s. It can be seen that only the first, low frequency points can be fitted. The corresponding wavevector range is located in the crossover region between the two limiting regimes of the theory, *i.e.* the tension-dominated regime on the lower q_s side, and the bending rigidity-dominated regime, on the higher q_s side. In the first one, the spectrum is described by the limit expression obtained from (4) for $\sigma \gg \kappa q^2$:

$$\lim_{\kappa \to 0} \langle h_{q_s}^2 \rangle \; = \; L \frac{k_BT}{2\sigma} \, q_s^{-1} \tag{5}$$

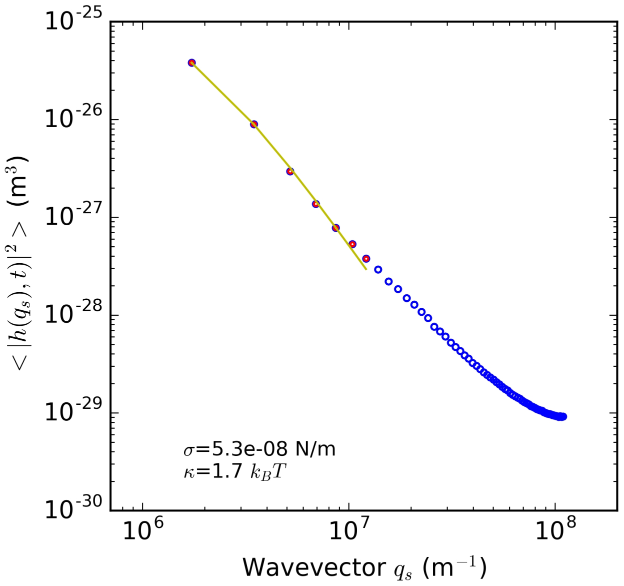

Fig. 3 : Average fluctuation spectrum (○). Best fit (line) is for the first seven wavevectors values (corresponding tension and bending modulus given as inset). Experimental points start deviating from the theoretical fit for *circa* $q_s = 10^7$ m^{-1}.

In the second one, the spectrum can be extracted from the theory for a low enough value of the internal tension of the membrane, *i.e.* for $\sigma \ll \kappa q^2$ in eq(4):

$$\lim_{\sigma \to 0} \langle h_{q_s}^2 \rangle \; = \; L \frac{k_BT}{4\kappa} \, q_s^{-3} \tag{6}$$

Deviation of the experimental spectrum from the q^{-3} slope at high q_s has been described elsewhere,? in the case of video imaging. Due to a finite, non zero value of the aperture time of the shutter of the camera, fluctuations with a lifetime shorter than this integration time are not correctly fitted. Although images were here obtained with a scanning method, due to the confocal technology, leading possibly to subtile, hard to derive effects on the fluctuation spectrum, we can however, as a first estimation, compare our frame acquisition rate of 172 im./sec (i.e. τ_{acq} = 5.8 ms) to the fluctuation lifetime τ_m given by? :

$$\tau_m(q)^{-1} = \left(\frac{1}{4q\eta} \right) \, (\sigma q^2 + \kappa q^4) \tag{7}$$

Under the condition of negligible internal tension on one side, or high internal tension on the other side, we get the corresponding expressions of the wavelength thresholds:

$$q_\kappa^C = \sqrt[3]{\frac{4\eta}{\kappa \tau_{acq}}} \quad \text{and} \quad q_\sigma^C = \frac{4\eta}{\tau \sigma} \tag{8}$$

with $\eta = 0.001$ kg/m·s the viscosity of the buffer, that reads $q_\kappa^C = 3.5\ 10^6$ m^{-1}, and $q_\sigma^C = 1.3\ 10^7$ m^{-1} respectively, defining a range that includes the wavevector value at which de-

viation between experimental and theoretical spectra starts (SI.Fig. 3).

Clearly our experimental conditions (i.e. mainly reduced observation field and low time resolution) provide partial data as far as spectral analysis is concerned. However, fitting (SI.Fig. 3) reads a value of the membrane bending rigidity $\kappa = 1.7\ k_BT$, *i.e.* in qualitative agreement with that already measured elsewhere.[?]

1.3 Autocorrelation analysis

Autocorrelation analysis

Correlation functions serve as a natural quantity for describing contour fluctuations that are the results of cooperative phenomena within the membrane and its environment. For a given membrane contour, imaged at time t and defined by its distance function $h(\theta,t)$ to the average membrane position (1), we can write the angular correlation function as[?] :

$$A(\theta,t) = \frac{1}{\langle R\rangle^2(\theta_{max}-\theta_{min})}\int_{\theta_{min}}^{\theta_{max}-\theta} h(\phi+\theta,t)\ h(\phi,t)\,d\phi \tag{9}$$

that corresponds here to:

$$A(\theta_n,t) = \frac{1}{128\langle R\rangle^2}\sum_{i=0}^{127-n} h(\theta_i,t)\ h(\theta_{i+n},t) \tag{10}$$

with $\theta_n = \theta_{min} + n\Delta\theta$ since each membrane contour was expressed as a set 128 $h(\theta,t)$ values. While $A(\theta_n,t)$ characterizes a single contour, its time average $\bar{A}(\theta_n)$ is expected to contain general informations about the processes governing membrane shape fluctuations. We thus define the time average over the full sequence of $\mathbf{N} = 1276$ images of Movie ESI.M16 as:

$$\bar{A}(\theta_n) = \frac{1}{\mathbf{N}}\sum_{i=0}^{\mathbf{N}-1} A(\theta_n,t_i) \tag{11}$$

As well explained in previous work,[?][?] instantaneous membrane undulations around an average, spherical shape, can be expressed by the variable $u(\vartheta,\varphi)$, *i.e.* the distance between the membrane and the average membrane position in the (ϑ,φ), spherical representation:

$$u(\vartheta,\varphi,t) = \sum_{l\geq 0}\sum_{n=-l}^{l} u_{l,n}(t)\ Y_{l,n}(\vartheta,\varphi) \tag{12}$$

with $Y_{l,n}(\vartheta,\varphi)$ the Bessel spherical harmonics, the $u_{l,n}$ corresponding to their amplitudes. Note that previously defined height is $h(\theta,t) = u(\vartheta = \pi/2,\theta)$. In the present case, only the intersection of the membrane with the 2D, focus plane of the objective of the microscope is observed. Thus, the angular autocorrelation function defined above can be expressed as:

$$A(\theta,t) = \sum_{l\geq 0}\sum_{l'\geq 0}\sum_{n\neq 0} u_{l,n}(t)\ u^*_{l',n}(t)\ Y_{l,n}(\frac{\pi}{2},\theta)\ Y^*_{l',n}(\frac{\pi}{2},\theta) \tag{13}$$

Averaging over time enables to simplify (13) by considering that $\langle u_{l,n}(t)\ u^*_{l',n}(t)\rangle = \langle |u_{l,0}|^2\rangle\delta_{ll'}$, thanks to the equipartition theorem.[?] We thus end up with the expression of the average autocorrelation function:

$$\begin{aligned}\bar{A}(\theta) &= \sum_{l\geq 0}\langle |u_{l,0}|^2\rangle \sum_{n=-l}^{l} Y_{l,n}(\frac{\pi}{2},\theta)\ Y^*_{l',n}(\frac{\pi}{2},\theta)\\ &= \sum_{l\geq 0}\langle \xi_l\rangle\ P_l(cos(\theta))\end{aligned} \tag{14}$$

with:

$$\langle \xi_l\rangle = \frac{2l+1}{4\pi}\ \langle |u_{l,0}|^2\rangle \tag{15}$$

and $P_l(x)$ are the Legendre Polynomials. For equilibrium fluctuations of small deviations around the spherical shape, it has been shown that, in absence of a natural curvature of the membrane, the $u_{l,0}$s obey[?] :

$$\langle |u_{l,0}|^2\rangle = \frac{k_BT}{\kappa(l-1)(l+2)[l(l+1)+\frac{\sigma R^2}{\kappa}]} \tag{16}$$

Both bending modulus κ and average membrane internal

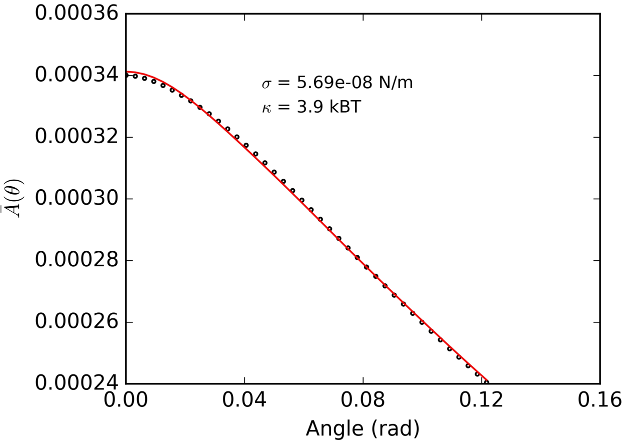

Fig. 4 : Spatial autocorrelation function (○). Best fit (line) reads the tension and bending modulus values given as inset.

tension σ can thus be obtained by fitting the experimental average autocorrelation distribution (11) by expression (14), using (15) and (16) (SI.Fig. 4). It is worth noting that $\langle\xi_0\rangle$ and $\langle\xi_1\rangle$ correspond to fluctuations of the radius and center of mass respectively of the round shape membrane.[?] They will thus be excluded from the analysis. Finally, index l in (14) is taken in the range $[3..l_{max}]$, where l_{max} is determined so that expression (14) converges with the right level of precision.

The autocorrelation analysis reads a value of the mem-

brane bending rigidity $\kappa = 3.9\ k_B T$ (SI.Fig. 4), closer to that given in litterature? than the one obtained with spectrum analysis (see Table 1 in the article).

2 ELECTRONIC SUPPLEMENTARY INFORMATION

Movie M1: LUVs stability at 20°C. Continous movie (3.3 im/s) of a typical $C_{10}E_3$ sample (3.33 g/L) at 20°C. Movie duration corresponds to 96 s; this movie is part of a longer movie (16 min), that shows no evolution of the system all over the period. Image size is 38.75×19.34 μm^2.

Movie M2: Tubes to LUVs transition. Continuous movie (13.8 im/s) of a $C_{10}E_3$ sample T-quenched at 30°C. The sample appears populated by membrane tubes, due to a strong shearing applied during the sample preparation (see Materials and Methods part in the article). Movie duration corresponds to 96 s; this movie is part of a longer movie (217 s). Image size is 38.75×19.34 μm^2. At the end of the sequence are tubes non visible anymore: only LUVs are present, similar to the ones in Movie M1. The whole movie reveals that LUVs remain present up to time 163 s. Only then on do LUVs start to evolve towards multilamellar objects, following the steps described in the article, and finally ending as sponge phase droplets. The observed kinetics of tubes-to-LUVs transition (typically 1.5 min) is way faster than at 20°C, a temperature at which typically 15 to 30 minutes were required.

Movie M3: Bretzel formation. Continuous movie (6.95 im/s) of a $C_{10}E_3$ sample T-quenched at 35°C. The movie shows the typical first stage of LUV structure transformation towards a multilamellar object and later on, eventually towards a sponge phase droplet (not shown). Movie duration corresponds to 14.5 s; this movie is part of Movie M8. Image size is 10.44×9.08 μm^2. Fig. 3 in the article shows six pictures taken from the movie.

Movie M4: T-quentching at 28°C. Continuous movie of a $C_{10}E_3$ sample quenched at 28°C. The movie is build from a time series of xyz-scan intensity averaged projections (38,75×38,75×12,7 μm^3). The movie starts a few seconds after the sample has been introduced in the temperature-controlled environment of the microscope. Total duration corresponds to 6 min 40 s.

Movie M5: T-quentching at 28°C. Continuous movie (13.74 im/s) of a $C_{10}E_3$ sample quenched at 28°C. Image size is 38.75×19.34 μm^2. The movie starts a few seconds after the sample has been introduced in the temperature-controlled environment of the microscope. Movie duration corresponds to 222 s.

Movie M6: T-quentching at 30°C. Movie (1.12 im/s) of a $C_{10}E_3$ sample quenched at 30°C. Movie duration corresponds to 251.2 s. Image size is 38.75×38.75 μm^2. The movie is extracted from a (z,t) scan, *i.e.* the original movie was a continuous sequence of scans over the z-coordinate (over 20.2 μm, 21 steps) over time. The extracted movie corresponds to one z position.

Movie M7: T-quentching at 30°C. Continuous movie of a $C_{10}E_3$ sample quenched at 30°C. The movie is build from a time series of xyz-scan intensity averaged projections (38,75×38,75×12,7 μm^3) recorded immediately after T-quenching. Total duration corresponds to 4 min 9 s.

Movie M8: T-quentching at 35°C. Continuous movie (13.86 im/s) of a $C_{10}E_3$ sample quenched at 35°C. Movie duration corresponds to 32.4 s. Image size is 38.75×19.34 μm^2.

Movie M9: Stable onion-like structures. Continuous movie (25.24 im/s) of a $C_{10}E_3$ sample T-quenched at 28°C, 6 min 20 s after quenching started. Stable multilamellar onions populate the cell. An automatic z-scanning has been imposed, covering a z-range of 20.17 μm (21 steps). Movie duration corresponds to 12.48 s. Image size is 38.75×19.34 μm^2.

Movie M10: Stable, big multilamellar structures. Continuous movie (1.05 im/s) of a $C_{10}E_3$ sample T-quenched at 28°C, 6 min 09 s after quenching started. Stable very large, multilamellar structures could be seen. Movie duration corresponds to 17.2 s. Image size is 91.18×45.54 μm^2.

Movie M11: Zoom on a stable multilamellar structure. Continuous movie (26.4 im/s) of a $C_{10}E_3$ sample T-quenched at 28°C, 14 min 16 s after quenching started. Movie duration corresponds to 24.8 s. Image size is 15.50×7.72 μm^2.

Movie M13: Fusion during T-quenching. Movie (1.12 im/s) of a $C_{10}E_3$ sample T-quenched at 30°C, 3 min 52 s after quenching started. Movie duration corresponds to 149.4 s. Image size is 38.75×38.75 μm^2. The movie is extracted from a (z,t) scan, *i.e.* the original movie was a continuous sequence of scans over the z-coordinate (20.2 μm over 21 steps) over time. The extracted movie corresponds to one z position.

Movie M14: Fusion during T-quenching. Movie (1.14

im/s) of a $C_{10}E_3$ sample T-quenched at 30°C, 5 min 19 s after quenching started. Movie duration corresponds to 37.7 s. Image size is 77.50×77.50 μm^2. The movie is extracted from a (z,t) scan, *i.e.* the original movie was a continuous sequence of scans over the z-coordinate (20.2 μm over 21 steps) over time. The extracted movie corresponds to one z position.

Movie M12: Big, stable intermediate structures at 28°C. Movie (1.16 im/s) of a $C_{10}E_3$ sample T-quenched at 28°C, 9 min 43 s after quenching started. Movie duration corresponds to 21.53 s. Image size is 77.50×77.50 μm^2. The movie is extracted from a (z,t) scan, *i.e.* the original movie was a continuous sequence of scans over the z-coordinate (20.2 μm over 21 steps) over time. The extracted movie corresponds to one z position.

Movie M15: Membrane dome. Continuous movie (27.6 im/s) of a $C_{10}E_3$ sample T-quenched at 28°C, 28 min 14 s after T-quenching started. Movie duration corresponds to 6.95 s. Image size is 19.38×19.38 μm^2.

Movie M16: Isolated bilayer. Continuous movie (172 im/s) of a $C_{10}E_3$ sample quenched at 28°C, 29 min 26 s after T-quenching started. Movie duration corresponds to 7.41 s. Image size is 3.87×1.92 μm^2.
